# Context dependency of trait repeatability and its relevance for management and conservation of fish populations

**DOI:** 10.1093/conphys/cow007

**Published:** 2016-03-23

**Authors:** S S Killen, B Adriaenssens, S Marras, G Claireaux, S J Cooke

**Affiliations:** af1 Institute of Biodiversity, Animal Health and Comparative Medicine, Graham Kerr Building, University of Glasgow, Glasgow G12 8QQ, UK; af2 IAMC-CNR, Institute for the Coastal Marine Environment, National Research Council, Località Sa Mardini, 09170 Torregrande, Oristano, Italy; af3 Université de Bretagne Occidentale, LEMAR (UMR 6539), Unité PFOM-ARN, Centre Ifremer de Bretagne, 29280 Plouzané, France; af4 Fish Ecology and Conservation Physiology Laboratory, Department of Biology and Institute of Environmental Science, Carleton University, 1125 Colonel By Drive, Ottawa, ON, Canada K1S 5B6

**Keywords:** Environmental effects, intraclass correlation, personality, phenotypic plasticity, reaction norm, temperature

## Abstract

Repeatability of behavioural and physiological traits is increasingly a focus for animal researchers, for which fish have become important models. Almost all of this work has been done in the context of evolutionary ecology, with few explicit attempts to apply repeatability and context dependency of trait variation toward understanding conservation-related issues. Here, we review work examining the degree to which repeatability of traits (such as boldness, swimming performance, metabolic rate and stress responsiveness) is context dependent. We review methods for quantifying repeatability (distinguishing between within-context and across-context repeatability) and confounding factors that may be especially problematic when attempting to measure repeatability in wild fish. Environmental factors such temperature, food availability, oxygen availability, hypercapnia, flow regime and pollutants all appear to alter trait repeatability in fishes. This suggests that anthropogenic environmental change could alter evolutionary trajectories by changing which individuals achieve the greatest fitness in a given set of conditions. Gaining a greater understanding of these effects will be crucial for our ability to forecast the effects of gradual environmental change, such as climate change and ocean acidification, the study of which is currently limited by our ability to examine trait changes over relatively short time scales. Also discussed are situations in which recent advances in technologies associated with electronic tags (biotelemetry and biologging) and respirometry will help to facilitate increased quantification of repeatability for physiological and integrative traits, which so far lag behind measures of repeatability of behavioural traits.

## Teaser

Animals show consistent differences in behaviour and physiology. Understanding these differences is vital for predicting the effects of gradual environmental change, such as climate change and ocean acidification. Here we review how such trait repeatability is relevant for conservation of wild fish.

## Introduction

‘Now, is it not true that if you want to address the interindividual variability, then you have to look at the intraindividual variability first? In fact, the only thing that remains beyond intraindividual variability is true interindividual variability.’ Reply by Peter Scheid to Bennett (1987).

Since the recognition that animal populations are composed of individuals that differ in their physiology and behaviour (Slater, 1981; Magurran, 1986; Bennett, 1987; Clark and Ehlinger, 1987), there has been growing interest in quantifying among-individual variability (Nespolo and Franco, 2007; Williams, 2008; Bell *et al.*, 2009). Such work has documented a large degree of among-individual variation for numerous physiological (e.g. metabolic rate and aerobic scope) and associated integrative traits (e.g. locomotion ability and susceptibility to environmental change) as well as behavioural traits (e.g. boldness, activity and aggression). This variation is crucial as the raw material for natural selection, but for a trait to be a determinant of individual fitness, it must also be heritable and stable (i.e. repeatable) over a time consistent with the intensity and nature of the selective pressure experienced. Indeed, trait repeatability has been suggested to set the upper limit for trait heritability (Falconer, 1981; Dohm, 2002; Dochtermann *et al.*, 2015).

Repeatability is often quantified as the proportion of variation within a population that is attributable to differences among individuals, as opposed to variation that occurs within individuals, although there are various ways to quantify repeatability in specific experimental conditions (Lessells and Boag, 1987; Bell *et al.*, 2009; Nakagawa and Schielzeth, 2010; Martin *et al.*, 2011; Biro and Stamps, 2015). Repeatability simultaneously depends on among-individual variance and within-individual consistency of the trait of interest. The available evidence suggests that many behavioural and physiological traits are indeed repeatable, although the magnitude of the variation observed and the degree of repeatability vary among traits, populations, species, life-history stages and the environment in which traits are measured (Nespolo and Franco, 2007; Williams, 2008; Bell *et al.*, 2009).

The majority of work quantifying trait variance and repeatability has been performed with the goal of understanding evolutionary processes in ecology, with few explicit attempts to apply repeatability and context dependency toward understanding management and conservation-related issues (Claireaux *et al.*, 2013). However, trait repeatability and the effects of environmental variables will play a large role in the evolutionary and plastic responses of species to a range of factors, including harvest-induced evolution, climate warming and ocean acidification. Certain aspects of environmental change may erode trait variation or repeatability and therefore the extent to which particular traits can be a target for selection (Dingemanse *et al.*, 2010; Killen *et al.*, 2013). Alternatively, environmental change may cause certain traits to become more important as targets for selection. Indeed, differences in repeatability among contexts may underlie differences in heritability and thus the magnitude of change potentially caused by directional selection (Dohm, 2002). Most work in the realm of conservation has focused on tracking population sizes and distributions and the quantification of biodiversity (Bellard *et al.*, 2012; Marras *et al.*, 2015a). However, without an understanding of how environmental factors affect among-individual variation and trait repeatability, we will be unable to anticipate more protracted evolutionary responses to anthropogenic environmental change that may in fact shape the genotypes and phenotypes of wild populations.

Here, we review work that has been done on trait variation and repeatability in fishes, and how these factors are affected by the environment. The key question we consider is as follows: is the best fish the best in every context or does the fittest fish within a population vary depending on the environmental conditions? Throughout, we discuss how these issues are relevant for management and conservation issues. We focus on fish because they are often used as models for research in both laboratory and field settings and because of the fact that they have often been at the forefront of research on repeatability of both behavioural and physiological traits (Huntingford, 1976; Mittelbach *et al.*, 2014). Freshwater and marine fish also provide numerous ecosystem services (Holmlund and Hammer, 1999) yet also face numerous threats (e.g. overharvest, habitat alteration, environmental change and invasive species) that make them of great interest to resource managers and conservation practitioners.

## Measurement of repeatability

### Methods for calculating repeatability

In its simplest form, repeatability is expressed as the proportion of total variance for a trait explained by between-individual differences, calculated as the intraclass correlation coefficient and denoted by *R* (Lessells and Boag, 1987; Nakagawa and Schielzeth, 2010); *R* can be calculated from variances partitioned using single-factor ANOVA or linear mixed models and estimates the agreement or reproducibility of absolute measurement values. This metric has been widely used in literature on repeatability of behaviour and physiology and lends itself well for systematic reviews or meta-analyses aimed at disentangling sources of variation in repeatability scores across studies (Nespolo and Franco, 2007; Bell *et al.*, 2009; Wolak *et al.*, 2012). In addition, *R* has the advantage that they enable direct comparison with their genotype-level equivalent, heritability (Dochtermann *et al.*, 2015).

The choice of repeatability metric can, however, be constrained by distributional assumptions and logistic limitations on the collection of sufficiently high numbers of repeated measures and resulting issues associated with poor statistical power (Martin *et al.*, 2011; Johnson *et al.*, 2015). This is particularly true for physiological traits, which are often invasive (e.g. lethal sampling of white muscle tissue or monitoring of cardiac output), time demanding (e.g. measuring metabolic rate) or expensive to perform large numbers of within-individual replicates. As such, researchers often use metrics other than *R* to estimate trait repeatability. A few studies have used Kendall's coefficient of concordance to estimate rank order stability among individuals (Sneddon *et al.*, 2006; Norin and Malte, 2011; Norin *et al.*, 2015). In addition, several studies have calculated individual consistency of traits with product–moment correlations (Pearson's *r* or Spearman's rank correlation coefficient, ρ (Hanson *et al.*, 2010; Marras *et al.*, 2011; Jornod and Roche, 2015). It should be noted that repeatability scored with these methods often reflects different aspects of score stability compared with that quantified using *R*. For instance, *r* estimates relative consistency between two scores independent of mean differences and is therefore more a measure of stability of relative differences among individuals, whereas *R* estimates agreement or reproducibility of absolute measurements (see also *R*_adj_ in the next paragraph). Thus, *R* will decrease with greater changes in mean values between separate measuring instances. More details on differences between these metrics and on underlying model assumptions can be found in the article by Nakagawa and Schielzeth (2010). Model assumptions should ideally be checked during the design stage of experiments to ensure that data collection efforts are not wasted.

### Repeatability in the presence of plasticity

**Figure 1:**
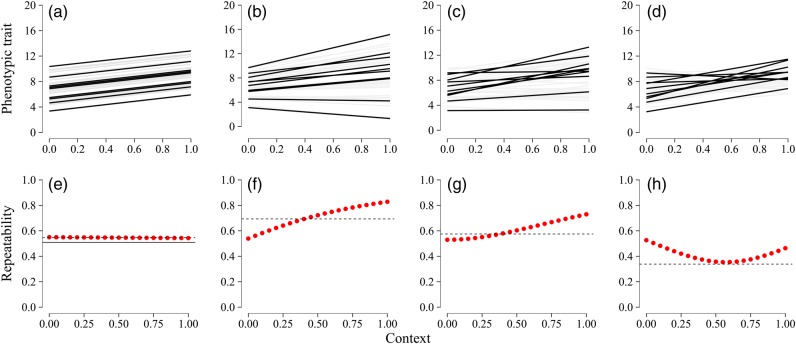
Using simulated data, we illustrate how plasticity to changing contexts interacts with three different metrics of repeatability discussed in the main text (*R*, *R*_adj_ and *R*_context_) and with cross-context correlations. Individual specific intercepts and slopes, fitted to simulated data using random regression methods, are presented in the top panels, whereas the bottom panels show *R* as continuous lines, *R*_adj_ as dashed lines and *R*_context_ with red dots. (**a**) and (**e**) represent a scenario in which individuals differ in mean traits (intercepts) but show equal increases of trait values with contexts (equal slopes). Given that individual rankings are maintained as contexts change, the cross-context correlation of trait values in context 0 vs. context 1 equals one in this scenario (*R* 0–1 = 1). Systematic changes in mean trait values across contexts further erode *R* values compared with *R*_adj_. Next, three scenarios are shown that differ in slope–intercept correlations while keeping all other parameters similar. In (**b**) and (**f**), individual slopes and intercepts show a strongly positive correlation (0.7), causing curves to fan out with little crossing among individual curves. This results in a gradual increase in *R*_context_ as context scores increase and a large cross-context correlation between context 0 and 1 (*R* 0–1 = 0.93). (**c**) and (**g**) show a scenario with zero slope–intercept correlation. This increases the incidence of crossing curves, tempers the rise of *R*_context_ and reduces the cross-context correlation of trait values (*R* 0–1 = 0.65). (**d**) and (**h**) show a scenario with strongly negative (−0.7) correlation between individual intercepts and slopes. In this scenario, the high incidence of curve crossings causes low cross-context correlations (*R* 0–1 = 0.13) and repeatabilities to reach a minimum in the context where most curves cross. Simulations are based upon a design in which 40 individuals are scored 15 times each across the full contextual gradient from 0 to 1. Parameters left unchanged in the simulated data are population-wide slope and intercepts (β intercept = 7, β context = 2) and between-individual differences in average traits (random intercept variance = 3). Between-individual differences in plasticity and residual error were similar in all panels (random slope variance = 5 and residual variance = 3) except for panels (a) and (e), where both were set to approximate zero. Curves are shown in black for 10 randomly selected individuals to enhance clarity of how each scenario affects crossing of individual curves, whereas remaining curves are plotted in light grey. All simulations and models were fitted using the package lme4 in R (Bates *et al.*, 2014; R Core Team, 2015). Full code in R for simulations, figures and calculation of context-specific repeatabilities and cross-environmental correlations is given in the supplementary material.

Many behavioural and physiological traits are labile within individuals and vary from day to day and among environmental contexts. Systematic changes in mean scores across measuring instances or contexts will erode *R* despite the maintenance of absolute differences between individuals. For instance, all fish might behave in an equally more bold manner when they grow or are repeatedly exposed to a specific experimental set-up [see Adriaenssens and Johnsson (2013) or Fig. 1a]. Owing to the impact of changes in mean values on *R*, many researchers have calculated *R* while accounting for such systematic changes within the fixed effects from mixed models (Adriaenssens and Johnsson, 2013; Harrison *et al.*, 2015). The resulting adjusted *R* values, or *R*_adj_, account for systematic trait changes across contexts and estimate repeatability as the agreement of individual differences at each measurement instance rather than absolute trait values (Nakagawa and Schielzeth, 2010). Figure 1e illustrates the difference between *R* (dashed line) and *R*_adj_ (continuous line) for a hypothetical example.

Often, however, plastic changes are not similar across individuals, and individuals differ in the slope of reaction norms across changing contexts. Boldness (Nagy *et al.*, 2010; Wilson *et al.*, 2011), activity (Biro and Adriaenssens, 2013) and aerobic metabolism (Careau and Garland, 2012; Metcalfe *et al.*, 2016), for example, can all exhibit high levels of individual differences in plasticity to mild temperature variation. Individuals can differ in plasticity to a wide variation of environmental factors or simply as a result of changes in traits over time as animals grow or learn about their environment. This individual phenotypic plasticity, or individual reaction norm variation, implies that individual variation will become context dependent, and consistency of traits can be highly impacted by environmental change (Nussey *et al.*, 2007; Dingemanse *et al.*, 2010).

As a result of individual plasticity, two aspects of individual consistency become of interest when we study individual differences in changing environmental contexts. First, we might examine the stability of individual trait differences within each environmental condition (within-context consistency). Second, we might want to determine how well individual trait measures in one environmental condition predict those in another (across-context consistency).

#### Within-context consistency

This application of repeatability refers to situations in which researchers quantify and compare repeatability in two or more sets of environmental conditions. For example, repeatability may be measured at two different temperatures. Several studies have performed separate ANOVA-based repeatability analyses on such data sets collected within different, but stable, contexts (Forsythe *et al.*, 2011; Sprenger *et al.*, 2012). Likewise, others have used separate product–moment, or Spearman, correlations to estimate context-specific trait stabilities when pairs of scores were available in each context (Maciak and Konarzewski, 2010; Adriaenssens and Johnsson, 2011). We also draw attention to recent publications showing how, for traits measured across a continuous environmental gradient, variance estimates from random intercept and slope mixed-effects models allow calculation of repeatability at any given context along the gradient (Brommer, 2013; Biro and Stamps, 2015). This context-specific repeatability (*R*_context_), also referred to as conditional repeatability (Nakagawa and Schielzeth, 2010), can provide a major tool for our understanding of how repeatability may change across environmental gradients, and potentially pinpoint environmental conditions that produce the greatest phenotypic diversity (bottom panels of Fig. 1, red dots). Figure 1 demonstrates how change in *R*_context_ along a gradient will largely depend on the extent of individual slope variation and how it relates to individual mean differences. When individuals show similar plasticity among contexts (Fig. 1a and e), there will be no change in among-individual variation or the rank order of a trait across the environmental gradient. In this situation, repeatability will also be unaffected by context. Yet, low or negative slope–intercept correlations tend to affect the order of individual scores across contexts greatly (see Fig. 1 for different scenarios). The end result is that *R*_context_ may change drastically depending on the conditions in which it is measured.

A current shortcoming in our interpretation of studies reporting within-context repeatability is that studies rarely investigate specifically whether separate repeatability scores are indeed statistically different. Note that observing significant repeatability in one context compared with another is not enough to reach this conclusion. As such, Fisher's Z transformations can be used to compare correlation coefficients statistically (Bell and Sih, 2007; Dingemanse *et al.*, 2007; Adriaenssens and Johnsson, 2013). Alternatively, one could specifically compare *R* scores by confidence/credibility interval overlap (Sprenger *et al.*, 2012) or using likelihood ratio tests (see supplement to Dingemanse and Dochtermann, 2013).

#### Across-context consistency

This application of repeatability occurs when researchers measure a trait on the same individuals in multiple environmental contexts (e.g. different temperatures, levels of oxygen availability and seasons) and then calculate one measure of repeatability across all measures. In balanced designs with one measure in each context, product–moment correlations or ranked alternatives have often been used to estimate the consistency of traits across contexts (Norin and Malte, 2011; Svendsen *et al.*, 2014; Taylor and Cooke, 2014). Others have calculated *R* from data collected in different contexts using ANOVA or simple random intercept mixed models without fitting individual slope variances accounting for individual differences in plasticity (Forsythe *et al.*, 2011; Killen *et al.*, 2012a, 2014). In this case, *R* describes the proportion of variance attributed to between-individual differences despite large changes to the measurement context between measuring intervals. It should be noted that repeatability calculated using methods not accounting for the individual variation in slopes will often deviate strongly from repeatability at any one context and are therefore limited in their ability to predict among-individual differences (Fig. 1; Schielzeth and Forstmeier, 2009; Brommer, 2013).

Alternatively, across-context correlations can be measured using the same mixed models used for calculation of *R*_context_ estimates. Across-context correlations estimate the extent to which rank orders of individual trait values are maintained when the context changes. They will equal one when slopes are equal among individuals (Fig. 1a and e) and approximate zero if there is no association between individual ranks in one context and another (Fig. 1d and h). If the context is measured on a continuous scale, and assuming sufficient replication across the full extent of the contextual gradient, across-context correlations can be estimated between each set of two contexts along the gradient, allowing for very detailed predictions about how trait values in one context predict those in another. See supplementary material for code in R to simulate Fig. 1 and calculate both *R*_context_ and across-context correlations. Currently, across-context correlations and *R*_context_ are available for only a handful of studies on fish (Biro and Adriaenssens, 2013; Harrison *et al.*, 2015) but are likely to provide a powerful tool for future field studies in which environmental variables (e.g. temperature) can be measured as a continuous variable.

Indeed, researchers have used a wide variety of metrics to measure individual consistency and often do this under the same heading of ‘repeatability’. In what follows, we aim to specify, where possible, which metric has been used whenever discussing studies illustrating variation in individual consistency (Pearson's *r*, Spearman's ρ, Kendall's coefficient of concordance, *R*, *R*_adj_ or *R*_context_).

## Potential confounding factors

There are a range of logistical and biological factors that need to be considered when estimating the repeatability of traits. This is especially true when traits are scored across different environments. Even in the absence of an obvious environmental gradient, time itself will by definition vary across measurement periods and so numerous factors intrinsic and extrinsic to the organism may change throughout the experiment that must be considered and controlled for when possible (Biro and Stamps, 2015). Here, we discuss some of these confounding factors and how they may be especially relevant when attempting to quantify variation and its repeatability in fish.

### Testing procedure over time

As repeatability introduces a time dimension, one must be sure to minimize time-dependent changes in sources of measurement error. An example in this regard can be observed when testing the repeatability of maximal and standard metabolic rates. As the organism will be likely to grow between measurements used to estimate repeatability, this will affect the ratio of respirometer chamber volume to organism volume (Svendsen *et al.*, 2016). The obvious solution is to increase the size of the experimental set-up as the animal grows, but this is in turn likely to affect the pattern of the measurement errors in an unpredictable manner. For instance, in aquatic respirometry, background bacterial oxygen consumption is a significant source of measurement error. Adjusting the size of a respirometry set-up also implies changing the magnitude of that error because the total volume of water in the set-up, as well as the internal surface area of that set-up, will be affected, with consequences in terms of bacterial biomass.

While changing an environmental factor of interest, researchers also need to be careful to control all other aspects of the environment that may confound the estimate of repeatability. Some of these interacting factors are, however, subtle and not easily identifiable. For example, improperly accounting for seasonal and circadian effects on animal physiology, morphology, metabolism and/or behaviour can obscure attempts to quantify repeatability. Properly including such factors in subsequent statistical analysis may be challenging. However, an advantage of mixed modeling approaches approaches for calculating repeatability is that, if they are able to be quantified, some of these confounding factors (e.g. time of day) can be included as fixed or random factors (Nakagawa and Schielzeth, 2010; Brommer, 2013).

In general, a specific type of measuring error can occur when not all fish are measured in the same environmental conditions. This can artificially inflate repeatability scores and result in so-called ‘pseudo-repeatability’ (or pseudo-personalities in the case of behavioural traits; Dingemanse and Dochtermann, 2013). Pseudo-repeatability can be particularly difficult to account for in wild roaming fish, where differences in food access, home range temperatures or positioning within social hierarchies may increase among-individual variation in behaviour and physiology. The existence of such ‘micro-niches’ in natural settings has also been suggested to explain higher observed repeatability in studies in the wild in comparison to laboratory studies, where environmental conditions are readily controlled (Bell *et al*., 2009).

### Ageing, habituation and physiological states

Growth and ageing, and associated rearranging of phenotypic architecture, are processes that are likely to blur repeatability. Likewise, effects of domestication must also be taken into account. Numerous anecdotal reports, but far fewer published ones, document the change in performance of wild animals as they familiarize themselves with laboratory conditions that have an optimized food supply and absence of predators (Adriaenssens and Johnsson, 2009). Habituation during multiple trials in behavioural assays also tends to turn responders into non-responders when examining, for instance, stress responses, and vice versa, such as when assessing whether individuals are bold–shy or proactive–reactive (Bell and Sih, 2007). Experimental work has also demonstrated apparent decreases in the repeatability (Spearman's ρ) of metabolic rate in fish over time (Norin and Malte, 2011), but variation in physiological traits could in theory be affected by laboratory holding of animals in homogeneous conditions. A challenge is to understand how such findings apply to long-term repeatability of physiological traits in the wild. Interestingly, sprint performance in blacknose dace (*Rhinichthys atratulus*) is repeatable when fish are held in the laboratory in high-flow conditions but not when held in more benign static-flow conditions (Spearman's ρ; Nelson *et al.*, 2008, 2015).

Extreme examples of temporal changes occur when multiple measures are made on fish as they transition between life stages or important life-history events. The effects of such changes on trait repeatability (*R*) can be drastic (e.g. sex change in hermaphroditic fish; Sprenger *et al.*, 2012) and should be avoided if this is not the specific focus of the research. Recent and long-term feeding history can also affect fish locomotory capacity (McKenzie *et al.*, 1995; Martinez *et al.*, 2002; Killen *et al.*, 2014), metabolic rate (Van Leeuwen *et al.*, 2012; Killen, 2014), activity (Krause *et al.*, 2000; Killen *et al.*, 2011), hormonal status (Cook *et al.*, 2012) and sociability (Krause, 1993; Killen *et al.*, 2016); therefore, consistency of diet is essential between trait measurement periods. Related to this set of factors, any differences in body size between measurement periods may confound estimates of trait variability and repeatability because of the allometric effects of body size on many behavioural and physiological traits (Glazier, 2005). Differences in body size among individuals will generate among-individual variation in the trait of interest. Depending on the research goals, investigators must carefully consider whether they want to include this source of variation in their estimates of repeatability, because not correcting for differences in size between measurement periods and among individuals will increase estimates of trait variation and repeatability.

The above-mentioned changes in testing procedures, traits and physiological states can be at the root of higher observed repeatability for short-term studies in comparison to studies with long intervals between repeated measures (Bell *et al*., 2009). Statistical methods have recently been developed for long-term studies with sufficient longitudinal data to estimate short-term repeatability vs. long-term repeatability within the same data set and will be likely to provide a greater insight into how these processes shape individual differences (Araya-Ajoy *et al.*, 2015).

### Source of animals for study and collection bias

The use of wild vs. cultured fish may affect estimates of repeatability in a variety of ways. For example, domesticated strains may possess lower levels of among-individual trait variance, thus reducing repeatability (*R*; Bell *et al.*, 2009). Wild animals, on the contrary, may show higher among-individual variance, which may also increase repeatability (Bell *et al.*, 2009). For studies using wild fish, there is a particular danger that variation in parasitic load could generate a substantial degree of among-individual variation that could inflate estimates of repeatability, whether the measures are performed in the laboratory or in the field. There is little known about the balance of these effects in fish, but Bell *et al*. (2009) found that estimates of repeatability across taxa seem to be higher in the wild than in the laboratory. White *et al*. (2015) observed within-context repeatability (Pearson's *r*) when juvenile Ambon damselfish (*Pomacentrus amboinensis*) were tested for a range of behavioural traits (e.g. activity and boldness) either in the laboratory or in the field (White *et al.*, 2015). Interestingly, however, this species showed low across-context repeatability (*R*) when the same individuals were tested in the laboratory and then again in the wild (White *et al.*, 2013). Adriaenssens and Johnsson (2011) provide experimental evidence of higher repeatability (Spearman's ρ) of exploratory behaviour in wild brown trout compared with those that were hatchery reared. For studies using wild fish, another important consideration is collection bias. Some individuals may be more vulnerable to sampling gears within a wild population (e.g. angling, capture by trap or trawl), and therefore an experimental population collected from the wild using only a single method may show artificially low among-individual variability for traits related to vulnerability to capture. More work is needed to understand how the use of wild vs. cultured fish may influence estimates of repeatability in different situations so that researchers can understand better how these results may translate to natural scenarios.

All of these issues are of foremost importance in cases where an environmental assessment is required, such as following the spill of a contaminant. In such cases, a population collected from an uncontaminated site is classically used as a control and is compared with the exposed population. This approach assumes that the observed among-population difference in the trait of interest is fully attributable to the spilled contaminant and that this effect is the same across the population affected by the spill. Unfortunately, this assumption is generally false and results in inappropriate conclusions regarding the impact of the spill. The effects of environmental influences on trait variability and repeatability on observations very clearly preclude direct comparison of populations with different environmental histories, including diet, and life-history trajectories. Failure to comply with a precautionary approach in this regard is likely to generate false-positive or false-negative effects when performing environmental assessments.

## Environmental contexts affecting trait variation and repeatability

The aquatic environment of fish can vary over time, inducing responses from gene to whole-animal levels (Johnston and Wilson, 2006). Environmental variables such temperature, oxygen availability and pH can affect the amount of variability within a population and, perhaps, the degree to which a physiological or a behavioural trait is repeatable. Furthermore, each of these factors may be susceptible to alteration via anthropogenic disturbance.

### Temperature

Temperature is among the most studied environmental factors affecting the physiology and behaviour of fish and generally of the greatest concern given the looming threat of climate change on aquatic habitats. The majority of previous work has focused on the consistency, or repeatability, of individual behaviour and physiology within a constant temperature, whereas the study of the possibility that trait repeatability can be maintained across different temperatures has received surprisingly little attention. For mosquitofish (*Gambusia holbrooki*), individuals differ in average activity levels, although repeatability of activity is a complex function of temperature and time since isolation, leading to approximately 2-fold differences in repeatability (*R*_context_) scores across time and thermal regime (Biro and Adriaenssens, 2013). Norin *et al*. (2015) found that standard and maximal metabolic rates of juvenile barramundi (*Lates calcarifer*) were repeatable (Pearson's *r*) across a 6°C increase in temperature, whereas aerobic scope was not significantly repeatable with the same temperature increase. Claireaux *et al*. (2007) found that sprint performance in the European seabass was repeatable (Spearman's ρ) when fish were acclimated to 12°C and then to 22°C (Claireaux *et al.*, 2007). Among the few studies that have examined trait repeatability in wild fish in a natural setting, several have considered the effects of daily and seasonal shifts in behaviour on repeatability of movement patterns, which would include thermal effects (Hanson *et al.*, 2010; Taylor and Cooke, 2014; Harrison *et al.*, 2015). Taylor and Cooke (2014), for instance, quantified movements of individual radio-tagged bulltrout (*Salvelinus confluentus*) in the wild and showed high rank-order consistency (Spearman's ρ) of mean movement distance across seasons and time of day. Cook *et al*. (2011) found repeatability in the glucocorticoid response in wild largemouth bass (*Micropterus salmoides*), but only after correction for strong effects of ambient temperature (Cook *et al.*, 2011).

### Oxygen availability

Aquatic environments exhibit extreme variation in the partial pressure of dissolved oxygen through time and space. Evidence suggests that the frequency and severity of hypoxic events in aquatic ecosystems has been worsening because of anthropogenic activities and eutrophication along waterways (Diaz and Rosenberg, 2008). Decreased oxygen availability can strongly influence the physiology and behaviour of aquatic breathers, and hypoxia is considered to be the most important environmental factor limiting aerobic metabolic scope in fish (Fry, 1971; Claireaux *et al.*, 2000). Studies at the intraspecific level show that hypoxia does not cause drastic changes to the extent of variation of standard metabolic rate or repeatability in spined loach (*Cobitis taenia*; Pearson's *r*; Maciak and Konarzewski, 2010). Another study demonstrated that the gulf killifish (*Fundulus grandis*) decreases metabolic rate during hypoxia but that repeatability is maintained (Pearson's *r*; Virani and Rees, 2000). Killen *et al*. (2012a) found that in juvenile European seabass, the tendency to take risks showed low repeatability across a gradient of oxygen availabilities (*R* and Spearman's ρ), probably because variation in spontaneous swimming activity was affected by the tendency of some individuals to perform aquatic surface respiration in hypoxic conditions. Joyce *et al*. (2016) examined the long term repeatability (18 months) of hypoxia tolerance in the European seabass and found that variability in whole-animal hypoxia tolerance was explained by interindividual variance in cardiac hypoxia tolerance. In hypoxic environments, variation in reaction norms to oxygen availability among individuals could conceivably put some individuals at a higher risk of adverse effects or mortality via predation in situations where activity increases in response to hypoxia.

### Food availability

Food availability and feeding history have a range of effects on fish physiology and behaviour (Wang *et al.*, 2006) as well as among-individual trait variation and repeatability. Periods of food deprivation are common for many fish species while overwintering or during periods of eutrophication during summer months when prey can become patchily distributed. Conditions of low food availability can increase among-individual variation in risk taking while foraging, and repeatability (Pearson's *r*) of risk-taking tendency appears to be maintained with a 1 week period of food deprivation in European sea bass (*Dicentrarchus labrax*; Killen *et al.*, 2011). Longer-term food deprivation seems to affect some forms of locomotory activity in fishes, especially anaerobic sprint-type swimming. However, effects on repeatability of sprint swimming during starvation are mixed. Martinez *et al*. (2002) found that the rank order of sprint performance was maintained in Atlantic cod (*Gadus morhua*; Spearman's ρ) after periods of starvation and refeeding, whereas Killen *et al*. (2011) found that a period of feeding caused repeatability (*R*) in sprint performance to decrease in European sea bass. It is possible that the direct effects of starvation and diet on aspects of muscle physiology, metabolism and energy stores may vary among individuals, leading to variable individual reaction norms. This effect may be exacerbated by the effects of compensatory growth trajectories during refeeding, which could further alter repeatability across feeding contexts (Metcalfe and Monaghan, 2001; Killen, 2014; Killen *et al.*, 2014).

### Carbon dioxide and pH

There is growing evidence that ocean acidification has significant and widespread impacts on marine life (Feely *et al.*, 2009). Elevated CO_2_ and reduced pH can greatly affect growth rate and survival in marine animals by altering their physiology and behaviour. The effects of reduced pH on a suite of physiological and behavioural variables and their level of variation have been largely studied in marine calcifiers, whereas in comparison the effects on marine fishes are poorly understood. Recent work showed contrasting results on the consistency of variation in physiological traits. Although Munday *et al*. (2009) found that larval fish exhibited considerable variation in the olfactory system responses to acidification (i.e. 700 ppm CO_2_), Cripps *et al*. (2011) did not find an increase in individual variation in olfactory sensitivity at a similar CO_2_ concentration (650 ppm). There have been documented effects of reduced pH on a number of ecologically relevant behaviours in fishes, including predator detection and avoidance (Ferrari *et al.*, 2012, 2015; Dixson *et al.*, 2015), but much more work is needed to establish whether such effects are repeatable at the individual level and the degree to which individuals show variation in reaction norms in response to CO_2_ exposure. Our lack of knowledge regarding how trait repeatability is affected by elevated CO_2_ and decreased pH is a currently a crucial factor restricting our ability to predict how aquatic organisms will evolve in response to acidification in marine and freshwater environments.

### Flow

There are a number of anthropogenic environmental disturbances that alter water flow patterns, including the construction of physical structures (e.g. dams, weirs and locks) as well as climate-induced changes in river flow volumes or oceanic currents. Increasing flow is known to affect the spatial positioning of individual fish within swimming schools, although individual spatial preference within groups does show a large degree of repeatability within flow speeds that allow aerobic steady-state swimming (Killen *et al.*, 2012b; Marras *et al.*, 2015b). Repeatability of sprint performance of blacknose dace (*Rhinichthys atratulus*) has been observed to be higher in high-flow conditions than when individuals are held in static water (Spearman's ρ; Nelson *et al.*, 2008, 2015). In the wild, the migration speed of individual sockeye salmon (*Onchorynchus nerka*) shows highest repeatability along non-turbulent river sections but drops near sections with heavy turbulence (Spearman's ρ; Hanson *et al.*, 2008).

### Pollutants

The available evidence suggests that individual fish may vary in their sensitivity to environmental pollutants. Kolok *et al*. (1998) observed that exposure to sediments with inorganic contaminants could alter the degree of among-individual variation in critical swimming speeds and repeatability in fathead minnows (Kolok *et al.*, 1998). Claireaux *et al*. (2013) documented that exposure to oil and oil dispersant had no effect on repeatability (Pearson's *r*) of hypoxia tolerance in juvenile sea bass (*Dicentrarchus labrax*), but both decreased repeatability of sensitivity to thermal stress in standardized challenge tests.

### Biotic factors

There are also biotic environmental influences that may affect estimates of trait variability and repeatability. Parasitic infection, for example, has been shown to alter traits such as boldness and activity level in fish, generating variation in these traits and thus affecting repeatability (*R*; Hammond-Tooke *et al.*, 2012). In a conservation context, changes to habitats that alter parasite communities may therefore change the degree of behavioural or physiological trait variation within fish populations. Social hierarchies in the wild and in the laboratory may also shape the behaviours and physiology of individual fish to increase trait variation and repeatability. For example, dominant fish can exhibit increased aggression and an increased metabolic rate associated with activity and the stress of hierarchy maintenance (Sloman *et al.*, 2000; Killen *et al.*, 2014). Changes to population density or habitats that destabilize social hierarchies may therefore alter social effects on individual trait variation and the repeatability of traits. Predator regime can also affect traits such as boldness (Archard *et al.*, 2012), suggesting a degree of community-level modulation over phenotypic expression that could be disrupted by various forms of anthropogenic environmental change. The effects of predator regime on trait variability and repeatability remains largely unexplored, but exposure to predator odour appears to decrease repeatability of boldness, activity and aggression in the common bully (*Gobiomorphus cotidianus*; Hammond-Tooke *et al.*, 2012).

## Beyond ‘mean’ repeatability: individual predictability

Low repeatability, and thus high within-individual variation, limits our power to infer underlying individual phenotypes accurately from any single physiological and behavioural measure. Correspondingly, there has recently been an increase in within-individual sampling effort (Bell *et al.*, 2009; Mittelbach *et al.*, 2014). We argue that the current emphasis on characterizing individuals only on a gradient of mean individual behaviour or physiology (e.g. from bold to shy or from low to high standard metabolic rate) leaves some biologically relevant information in repeated measures unexplored. For example, differences in within-individual variance or predictability may be as important for our understanding of population resilience as individual differences in mean phenotypes (Stamps *et al.*, 2012; Westneat *et al.*, 2015). Indeed, the degree to which individuals show variation in behaviour or physiological responses may itself be repeatable and relevant for determining evolutionary responses to environmental change. A key question is whether an animal that shows high within-individual variability in behaviour and physiology will be more or less vulnerable to environmental change. On the one hand, such an individual may be less vulnerable if variability allows it to cope with differing conditions. On the other hand, if expressing variability is essential for survival, then any change to the environment that constrains this variability would make individuals more vulnerable. Understanding the role of within-individual variation, how this is affected by the environment, and its role in population responses to environmental change is at present an open area for research.

So far, only two studies have reported individual differences in predictability for fish behaviour. Damselfish (*Pomacentrus wardi*) show within-individual variability in latency to emerge following a stimulus, and the magnitude of this variation varies among individuals (Stamps *et al.*, 2012). In mosquitofish, individual differences in within-individual variation in activity consistently manifest over long periods of time, but fish become more predictable as they are kept in isolation when in captivity (Biro and Adriaenssens, 2013). Differences in individual predictability may have an important role for fish conservation by influencing how individuals cope with varying environments. This may be especially true for physiological traits that tend to be buffered around a certain optimal value, for which individual predictability may serve as an indicator of individual health and, perhaps, ecosystem health in the face of anthropogenic stressors (e.g. blood pressure; Sandblom *et al.*, 2012). We suggest that studies investigating mean differences, plasticity and predictability in concert may prove fruitful for our understanding of how populations are impacted by anthropogenic change. Human activities may further alter selection pressures on heritable components of plasticity and predictability within the population and may shift the abundance of individual phenotypes, as has been observed for mean phenotypes. Yet, despite our long-held appreciation of the role of plasticity for population resilience (West-Eberhard, 1989; Oomen and Hutchings, 2015), the occurrence and ecological effects of such human-caused alterations of individual plasticity remain poorly studied.

Although useful information can be gained from comparing groups for predictability (Cleasby and Nakagawa, 2011), teasing apart different aspects of the phenotype between individuals (e.g. individual reaction norm slopes from intercepts, or individual variance from means) requires sufficient replication at the within-individual level (Dingemanse and Dochtermann, 2013; Cleasby *et al.*, 2015). The rise of new techniques that allow minimally invasive and repeated observations of physiological and behavioural traits will therefore be likely to play a large role in increasing our understanding of within-individual variation in both the laboratory and the field. Telemetry data, for example, may be ideal for quantifying how within-individual variance in movement patterns may be affected by temperature, food availability, population density or anthropogenic noise. Advances in intermittent-flow respirometry may also make it possible to study within-individual variance in metabolic rate in response to numerous environmental factors, an issue that has so far been neglected. Water-borne hormone assays may also allow for an increase in repeated sampling of individuals to provide information on within-individual variation in stress responsiveness and endocrine status (Ellis *et al.*, 2004). The use of high-throughput challenge tests is also a promising approach because it allows assessment of the variability and repeatability of integrated performance, such as swimming capacity, hypoxia tolerance and thermal sensitivity, in populations of hundreds of individuals (Castro *et al.*, 2013; Claireaux *et al.*, 2013).

## Outlook: fundamental and applied perspectives

The article by Bennett (1987) on interindividual variability as an ‘underutilized resource’ will long be heralded as a wake-up call for researchers in the realms of ecological and environmental physiology. Interestingly, at around the same time, research in ethology went through a similar transformation after several authors focused attention on among-individual differences (Huntingford, 1976; Slater, 1981; Magurran, 1986; Clark and Ehlinger, 1987). Together, these papers fuelled a large and growing body of work on trait repeatability in a variety of organisms, including wild fish (Williams, 2008; Bell *et al.*, 2009). There is currently a large research effort to integrate our understanding of individual behaviour and physiology (Cockrem, 2007; Careau *et al.*, 2008; Koolhaas, 2008; Biro and Stamps, 2010). There is also an awareness that environmental stressors can have effects on both behaviour and physiology (Killen *et al.*, 2013).

It is unclear whether Bennett and others could have predicted the extent to which their seminal work would shape research programmes for countless scientists, nor the extent to which the concepts they developed would have relevance to conservation and resource management. Here, we provide an outlook for the study of trait repeatability in wild fish (fundamental perspectives) as well as the ways in which such information is or could be relevant to resource managers and conservation practitioners (applied perspectives).

### Fundamental perspectives from the study of fish in the wild

The available evidence from fish suggests that repeatability of traits seems to be highly context dependent (Table 1). To date, most of this evidence has come from laboratory studies, but this is likely to be true particularly for wild fish that live in dynamic (e.g. seasonality, tidal cycles; see Koukkari and Sothern, 2007) and, often, unpredictable environments (Wingfield, 2003). Food availability and quality, competition, pathogen and parasite loads, predators, habitat quality and physiochemical variables (e.g. temperature and dissolved oxygen) are not static. Moreover, an individual fish is constantly changing as it ages and grows, transitioning through different life stages. Layered on top are human activities that may expose wild fish to multiple, potentially, novel stressors (Angelier and Wingfield, 2013) or contexts (Sih, 2013). All of these factors have the potential to influence the extent to which a given trait or suite of traits is repeatable. As more researchers become interested in quantifying trait repeatability in field studies, a major challenge will be to dissect the proportion of trait variance and repeatability that is a result of environmental effects vs. that which is intrinsic to individual animals. Wild largemouth bass, for example, appear to show intrinsic repeatability in the glucocorticoid response between years, but this is masked by the overriding effect of seasonal temperature fluctuations or other aspects of seasonality (Cook *et al.*, 2011). However, even the combined influence of genetic and environmental effects will be of interest because this will determine which phenotypes are ultimately exposed to selective pressures.

**Table 1:** Estimates of within-context and across-context repeatability in studies on fish

Species	Field/laboratory	Wild/Cultured	Trait	Environmental variable	Time span	Context^a^	Repeatability	Method	Reference
*Gambusia holbrooki*	Laboratory		Activity	Temperature and days since isolation	132 days maximum	24.4°C, 4 days	0.37	Single random intercept, random slope model	Biro and Adriaenssens (2013)
						24.4°C, 65 days	0.38		
						24.4°C, 132 days	0.55		
						26.3°C, 4 days	0.49		
						26.3°C, 65 days	0.3		
						26.3°C, 132 days	0.27		
*Lota lota*	Field	Wild	Movement	Season	2 years	Across context	0.98	Single random intercept, random slope model	Harrison *et al*. (2015)
						Summer	0.13		
						Winter	0.28		
			Vertical activity	Season		Across context	0.43		
						Summer	0.56		
						Winter	0.53		
			Vertical activity	Season		Across context	0.26		
						Summer	0.69		
						Winter	0.69		
*Salvelinus confluentus*	Field	Wild	Movement distance	Season	Several months	Across context	0.78	Spearman rank correlation	Taylor and Cooke (2014)
			Maximum distance			Across context	0.49		
			Movement distance	Time	Several hours	Across context	0.81	Spearman rank correlation	
			Maximum distance			Across context	0.62		
*Micropterus salmoides*	Laboratory		Critical swimming speed	Temperature	4 days	11°C	0.86	Spearman rank correlation	Kolok (1992)
						22°C	0.77		
*Dicentrarchus labrax*	Laboratory	Cultured	Emergence time	Before and after fasting	7 days	Across context	0.12	Pearson correlation	Killen *et al*. (2012a)
			Time out from cover			Across context	0.20		
			Activity			Across context	0.52		
*Liza aurata*	Laboratory	Wild	Position within school	Swimming speed	Several hours	10–20 cm s^−1^	0.36	Pearson correlation	
						10–30 cm s^−1^	0.31		
						20–30 cm s^−1^	0.78		
*Dicentrarchus labrax*	Laboratory	Wild	Emergence time	Varying levels of hypoxia	3 days	Across context	0.16	Intraclass correlation coefficient	Killen *et al*. (2011)
			Time out from cover			Across context	0.16		
			Activity			Across context	0.08		
*Oncorhynchus nerka*	Field	Wild	Migration speed	Flow		Ocean environment	0.27	Spearman rank correlation	Hanson *et al*. (2008)
						Turbulent river	0.13–0.18		
						Non-turbulent river	0.40–0.79		
	Field	Wild	Energy density	Spawning migration		Across context	0.98	Spearman rank correlation	
*Acipenser fulvescens*	Field	Wild	Spawning time	Season	8 years	Across context	0.42–0.56	Intraclass correlation coefficient	Forsythe *et al*. (2011)
			Spawning location			Across context	0.14–0.16		
*Micropterus salmoides*	Field	Wild	Daily distance travelled	Season	Several months to 2 years	Autumn	0.31–0.76	Spearman rank correlation	Hanson *et al*. (2010)
						Winter	0.51–0.72		
						Spring	0.19–0.51		
						Summer	0.75–0.92		
			Mean daily swimming speed			Autumn	0.09–0.62		
						Winter	0.65–0.70		
						Spring	0.25–0.52		
						Summer	0.55–0.91		
*Lepomis macrochirus*	Laboratory	Wild	Cortisol	Time	6 days	Across context	0.43	Intraclass correlation coefficient	Cook *et al*. (2012)
*Micropterus salmoides*	Field	Wild	Cortisol	Baseline	1 year	Baseline	0.06	Pearson correlation	Cook *et al*. (2011)
				Stress induced	1 year	Stress induced	0.19		
*Dicentrarchus labrax*	Laboratory		Sprint swimming speed	With and without fasting	45 days	Control	0.21	Intraclass correlation coefficient	Killen *et al*. (2014)
						Fasting	0.19		
				Compensatory growth	30 days	Control	0.51		
						Growth compensated	0.31		
*Gadus morhua*	Field	Wild	Sprint swimming speed	Intermittant periods fasting/feeding	Several weeks		0.48–0.54	Spearman rank correlation	Martinez *et al*. (2002)
			Phosphofructokinase				0.08–0.57		
			Lactate dehydrogenase				0.09–0.66		
			Cytochrome *c* oxidase				−0.65		
			Nucleoside-diphosphate kinase				0.16–0.57		
*Dicentrarchus labrax*			Hypoxia tolerance	Oil and dispersant exposure	2 months	Control	0.57–0.65	Pearson correlation	Claireaux *et al*. (2013)
						Oil exposed	0.71–0.77		
						Oil + dispersant exposed	0.60–0.71		
						Dispersant exposed	0.61–0.75		
			Temperature tolerance	Oil and dispersant exposure		Control	0.35–0.68		
						Oil exposed	0.73		
						Oil + dispersant exposed	0.77		
						Dispersant exposed	0.24–0.68		
*Gobiomorphus cotidianus*	Laboratory	Wild	Activity	Exposure to predator odour	3 weeks	Across context	0.28	Binomial generalised linear mixed-effects models	Hammond-Tooke *et al*. (2012)
					1 week	Control	0.77		
					1 week	Predator odour	<0.14		
			Aggression	Exposure to predator odour	3 weeks	Across context	0.27		
					1 week	Control	0.07		
					1 week	Predator odour	<0.14		
			Boldness	Exposure to predator odour	3 weeks	Across context	0.18		
					1 week	Control	0.41		
					1 week	Predator odour	<0.14		
*Dicentrachus labrax*	Laboratory	Cultured	Sprint swimming speed	Temperature (acclimation to 12 and then 22°C)	4 weeks	Across context	0.43	Spearman rank correlation	Claireaux *et al*. (2007)
*Rhinichthys atratulus*	Laboratory	Wild	Critical swimming speed	Exposure to training	40–50 days	Across context	0.35	Spearman rank correlation	Nelson *et al*. (2015)
			Sprint swimming speed			Across context	0.4		
*Pomacentrus amboinensis*	Both	Wild	Boldness	Arena size (small aquaria, large aquaria, field)	20 min	Across context	Low (exact value not presented)		
			Activity			Across context	Low (exact value not presented)		
			Bite rate			Across context	0.48	Pearson correlation	White *et al*. (2013)
*Pomacentrus amboinensis*	Laboratory	Wild	Latency to emerge	Laboratory vs. field	30 min	Laboratory	0.38	Intraclass correlation coefficient	White *et al*. (2015)
			Location			Laboratory	0.54		
	Field	Wild	Bite rate		30 min	Field	0.64		
			Activity			Field	0.69		
			Position in water column			Field	0.52		
			Aggression latency			Field	0.2		
			Aggesssion strikes			Field	0.2		
			Bite rate		3 days	Field	0.77		
			Activity			Field	0.62		
			Position in water column			Field	0.33		

a Included are studies that examined trait repeatability within or across multiple environmental contexts or that performed measures using fish in the wild across multiple seasons.

Identifying the context dependency of traits requires long-term study, particularly for long-lived organisms (i.e. many vertebrates). Time itself and its association with ontogeny, maturation and senescence would be highly relevant but is rarely studied in the context of repeatability given that most studies are of short duration. Aspects of habitat use, including depth, are also essential for understanding variation within populations, potential shifts in behaviour induced by environmental change, and variability among individuals in the ability to cope with such changes. Field studies will be essential in this regard, and there are a growing number of examples where researchers are using electronic tagging or marking of animals (e.g. with biologgers or biotelemetry devices that incorporate sensors; see Cooke *et al.*, 2004) to assess repeatability of behaviour and physiology through time and a range of environmental conditions.

In general, there have been fewer attempts to quantify within- and across-context repeatability for physiological traits in fishes compared with behavioural traits (Table 1). This is a crucial gap in knowledge, because an understanding of physiological mechanisms is key for predicting potential responses of species to environmental change (Horodysky *et al.*, 2015). There is also much more work needed on interactions among factors given that nature is inherently complex even in the absence of human environmental change and disturbance. Until recently, the analytical toolbox to quantitatively assess and evaluate cross-contextual repeatability has hampered such research on both behavioural and physiological traits in a natural setting. However, of late there have been a number of techniques developed to enable more sophisticated analysis of repeatability across contexts (Nakagawa and Schielzeth, 2010; Martin *et al.*, 2011; Brommer, 2013; Dingemanse and Dochtermann, 2013). The challenge for such analyses is the dependency on multiple repeated measures in each context and relatively large sample sizes. This has been an especially important hurdle for estimating repeatability of physiological traits, but recent advances in telemetry, respirometry and endocrine analysis will provide important insights in this area in coming years.

### Applied significance of repeatability in natural populations

From an applied perspective, there are many opportunities for exploring the importance of the context specificity of trait repeatability. For example, a major question in current studies examining the effects of climate change and ocean acidification is whether species will be able to adapt over the course of several generations to gradual environmental changes. To date, studies examining the effects of these aspects of global change have necessarily been performed over relatively short time scales (i.e. days to months of exposure to varying temperature or pH treatments), whereas in reality, wild populations will have decades or centuries to respond to such changes. An increased understanding of how trait variation and repeatability is affected by these factors will help us to gain a greater understanding of the capacity for adaption present in populations. Given the pervasiveness of human disturbance, evaluating the consistency of trait repeatability relative to other types of disturbance gradients would also be useful.

Moving forward, there are a number of research priorities that we regard as important for applying research into trait repeatability toward the conservation of wild fish populations. First is the need to look at more contextual variables. Of late there has been much focus on temperature given the imminence of climate change. As discussed in this review, however, there are a number of additional factors that may play a key role in affecting trait repeatability. Flow seems to be a particularly overlooked factor in this regard, particularly given the manner in which humans have harnessed and altered rivers. The observation that turbulent flow degrades the repeatability of migration speed (Hanson *et al.*, 2008) could have important consequences for local stock adaptation (Eliason *et al.*, 2011). Although stocks may be adapted to migrate to particular spawning grounds at specific times, sections of turbulent flow could act as crucial bottlenecks where among-individual variation and repeatability is collapsed, potentially overriding historical selection on traits that yield local adaptation. Such information could be used to improve the management of regulated rivers (e.g. understanding how fish respond to ramping flows, fishway use and success). Measures of trait repeatability in response to such measures could be used as concrete indicators of successful mitigation of turbulent or altered flow regime. In urban environments, increased runoff resulting from impervious surface cover can have dramatic effects on river and stream flow, with potential impacts on the repeatability of locomotory capacity in fish inhabiting these environments (Nelson *et al.*, 2008, 2015).

There are a number of other conservation issues that would benefit from further knowledge of trait repeatability among and within contexts. Behavioural traits such as individual sociability appear to be related to dispersal potential within fish species and, for invasive species, may influence which individuals with which specific traits disperse furthest and end up along the invasion front (Cote *et al.*, 2010; Chapple *et al.*, 2012). The reaction norms of individuals within an invasive population as they encounter different environmental conditions within a non-native habitat could determine whether or not an invasion is successful or which phenotypes within the invading population ultimately colonize new habitats. The potential confounds of captivity and domestication on trait repeatability could also have important consequences for conservation efforts involving stock enhancement with captive-reared fish. It is possible to select animals for a particular desirable trait in captivity (e.g. predator avoidance), but there is a need to understand whether that trait is genuinely repeatable in other contexts, such as those experienced in the wild.

We encourage research examining how environmental factors such as temperature and ambient oxygen level affect repeatability of integrative traits that are directly relevant for conservation. Greater emphasis on ecologically relevant events (e.g. repeatability of phenology such as timing of migration and breeding; Forsythe *et al.*, 2011) or traits that will have the greatest impact on conservation efforts or are most closely aligned with fitness would be an obvious priority. With regard to anthropogenic impacts on fish populations, this could include viewing vulnerability to angling (Philipp *et al.*, 2009) or commercial fishing (Diaz Pauli *et al.*, 2015; Killen *et al.*, 2015) as organismal traits unto themselves. It is plausible that the vulnerability of any single fish to capture may vary with the environment; the individuals most likely to be captured in one set of conditions may be least vulnerable in another (Killen *et al.*, 2015). Such effects would have important consequences for fisheries-induced evolution, and increased knowledge of how trait variability and repeatability are affected by shifting environmental conditions could in theory feed directly into management decisions. Where feasible, for example, management actions that spread fishing effort over a wider range of abiotic conditions could potentially reduce capture bias and preserve more phenotypic variation. Alternatively, efforts to fish in environmental conditions that produce the least amount of among-individual variability and repeatability would be expected to minimize the potential for selective effects on particularly vulnerable phenotypes.

One approach that we encourage for including repeatability estimates in applied research is to have researchers interact with managers or stakeholders when designing studies. For example, it would be sensible to ask them what traits they have observed to be repeatable and which contexts are most relevant for the system or issue. A logical approach may be first to determine whether there are repeatable traits in benign environments (again with input from the start from managers regarding which traits to focus on), starting with one to three traits. There may also be situations where it will be feasible to measure multiple traits between populations where nature is already applying experimental treatments (e.g. different temperatures along a latitudinal or altitudinal gradient). It is worth noting that efforts may be useless to managers if sample sizes are too small to infer anything biologically relevant. Given the rapidly advancing statistical techniques for assessing trait repeatability in various situations and experimental designs, we suggest that researchers consult with statisticians whenever possible when designing studies to determine how best to overcome the logistical difficulties of working in the field with wild animals while still providing useful information.

## Conclusions

We expect that in the coming years we will find additional examples where we think we understand a phenomenon, but things change drastically in another context. This will have important consequences, because shifts in environmental conditions brought on by human activities may change not only population abundances and distributions, but also which individual fish will gain a selective advantage in response to the prevailing selective forces. The individuals that have the greatest fitness in one set of conditions may be the least fit in another environment, or vice versa. Human-associated environmental change is therefore likely to affect evolutionary trajectories. Over shorter time scales, variation in trait repeatability among contexts could also affect our ability to transfer knowledge of physiology and behaviour gained in one context (e.g. the laboratory) to another.

Urgently needed is more work examining trait repeatability in the field and how repeatability of traits directly relevant for conservation will respond to various environmental stressors. We certainly want to emphasize the importance of studying wild fish in the wild (as opposed to in the laboratory), but mesocosm studies and laboratory-oriented experiments will continue to be important in the future because they enable researchers to manipulate the context systematically while imparting necessary controls. The most powerful approaches to understanding environmental effects on trait repeatability will be those that bridge the field and the laboratory and combine long-term observational studies with experimental manipulations.

## Supplementary material

Supplementary material is available at *Conservation Physiology* online.

## Funding

S.S.K. was supported by Natural Environment Research Council (NERC) Advanced Fellowship NE/J019100/1 and European Research Council Starting grant no. 640004. S.J.C. was supported by the Natural Sciences and Engineering Research Council (NSERC) and the Canada Research Chairs Program. B.A. was supported by a NERC standard grant NE/K00400X/1.

## Supplementary Material

Supplementary Data
